# Prognostic factors for medial open-wedge high tibial osteotomy with spacer implantation in patients with medial compartmental knee osteoarthritis

**DOI:** 10.1186/s13018-022-02934-8

**Published:** 2022-01-28

**Authors:** Fengkun Wang, Wenru Ma, Jinli Chen, Wenbin Cong, Yingze Zhang, Tengbo Yu, Yi Zhang

**Affiliations:** 1 Department of Sports Medicine, Affiliated Hospital of Qingdao University, 59 Haier Road, Laoshan District, Qingdao, 266000 Shandong Province China; 2 Department of Clinical Medicine, Qingdao University, Qingdao, Shandong Province China; 3 Department of Radiology, Affiliated Hospital of Qingdao University, Qingdao, Shandong Province China; 4 Department of Orthopedics, Affiliated Hospital of Qingdao University, Qingdao, Shandong Province China; 5 Department of Orthopedics, The Third Hospital of Hebei Medical University, Shijiazhuang, Hebei Province China; 6 Institute of Sports Medicine and Rehabilitation, Qingdao University, Qingdao, Shandong Province China; 7 Shandong Institute of Traumatic Orthopedics, Qingdao, Shandong Province China

**Keywords:** Medial opening-wedge high tibial osteotomy, Absorbable implants, Medial compartment osteoarthritis, Knee, Age factors, Kellgren–Lawrence grade

## Abstract

**Background:**

To identify medial open-wedge high tibial osteotomy (MOWHTO) prognostic factors with wedge-shaped spacer implantation (spacer-type MOWHTO) for varus medial compartment knee osteoarthritis.

**Methods:**

Patients who underwent spacer-type MOWHTO between August 2018 and September 2019 were prospectively enrolled in this study. Patients were divided into effective group and invalid group based on the Western Ontario and McMasters University Osteoarthritis Index (WOMAC) score one year postoperatively. The variables assessed at baseline and one year postoperatively including age, sex, body mass index (BMI), Kellgren–Lawrence (K–L) grade, hip–knee–ankle angle (HKAA), medial proximal tibial angle (MPTA), posterior tibial slope angle (PTSA), Blackburn–Peel index (BPI), duration of symptoms, and WOMAC score were compared. Prognostic factors were analyzed using logistic regression, and the corresponding odds ratios were also calculated.

**Results:**

A total of 104 patients were enrolled in the study protocol at one year postoperatively. The WOMAC score decreased from 72.39 ± 12.95 at baseline to 20.06 ± 12.96 at one year postoperatively. Univariate analysis revealed that the significant predictors of the WOMAC score were age > 70 years, BMI > 30 kg/m^2^, K–L grade IV, and pre-HKAA > 10° (*P* < 0.1 for all). Multivariable logistic regression analysis revealed that age > 70 (OR = 4.861) and K–L grade IV (OR = 6.590) were significantly associated with the higher WOMAC score at one year postoperatively.

**Conclusions:**

Spacer-type MOWHTO is an effective treatment for osteoarthritis with varus deformity. The prognostic factors for spacer-type MOWHTO are age and K–L grade.

## Background

Osteoarthritis (OA) is one of the major causes of mobility disorders and disability and affects over 10% of men and 13% of women older than 60 years [1, 2]. OA is approximately ten times more frequent in the medial knee compartment than in the lateral compartment, and varus (but not valgus) alignment increases the progression of knee OA and eventually leads to the need for total knee arthroplasty (TKA) [3–9]. Medial open-wedge high tibial osteotomy (MOWHTO) is a highly effective surgical procedure in younger patients with OA [5, 10–12]. It works by relocating the weight-bearing axis from the medial pathological compartment to the lateral healthy joint space. The main advantages of MOWHTO are joint preservation and minimal trauma. Moreover, MOWHTO achieves pain relief and improvement in knee function, which can postpone the need for TKA by 7–20 years [13–15].

MOWHTO is generally performed through an oblique incision on the medial side of the tibia while preserving the lateral tibial cortex [16, 17]. Zhang et al. recently reported a more minimally invasive and simple osteotomy in MOWHTO. This new type of MOWHTO used a novel absorbable wedge-shaped spacer composed of 30% β-tricalcium phosphate and 70% poly (lactic-co-glycolic acid) instead of a locking compression plate system [18]. Compared with conventional MOWHTO, this novel method has a lower cost, avoids the need for secondary surgery for fixation removal, and improves radiographic appearance and knee function during follow-up [18, 19]. Essentially, this type of absorbable spacer is a bone graft substitute that provides support in the open wedge without rigid internal fixation; however, the longitudinal stability of the cut tibia cannot be guaranteed. Zhang et al. also performed simultaneous proximal fibular osteotomy, which provides a better valgus orthopedic effect but may cause more complications or defects [18, 19].

Considering the advantages of this novel osteotomy method, we hypothesized that the prognostic factors for MOWHTO with spacer implantation (spacer-type MOWHTO) would differ from those for conventional MOWHTO with internal fixation. Thus, spacer-type MOWHTO may have more limited and strict indications than conventional MOWHTO. Additionally, it is unclear whether the results of spacer-type MOWHTO are significantly influenced by the pre- and postoperative radiographic axes. Therefore, it is necessary to define clear prognostic factors for evaluation prior to spacer-type MOWHTO. The objective of this prospective study was to identify the prognostic factors for successful spacer-type MOWHTO.

## Materials and methods

### Patients

Study approval was obtained from the research ethics committee (QYFYWZLL26146). From August 2018 to September 2019, a total of 111 spacer-type MOWHTO surgeries were performed by a single surgeon. Patient characteristics and surgical data were collected. All patients were asked for their consent to participate in the study before surgery. The inclusion criteria were as follows: (1) spacer-type MOWHTO performed during the aforementioned study period, (2) no history of knee surgery or trauma, (3) unilateral medial compartment knee OA with < 15° varus malalignment on weight-bearing full-leg anteroposterior radiographs, (4) K–L grade II–IV, (5) knee range of motion of more than 20° in extension and 100° in flexion, and (6) minimum follow-up of one year with clinical outcome evaluation. We excluded seven patients. Two patients required a plate to stabilize the tibia during the surgical procedures because of an unstable hinge fracture. Two patients needed reoperation because of tibial fractures due to a fall during the rehabilitation process. In addition, three patients were excluded due to being considered lost to follow-up.

### Surgical procedures

**Fig. 1:**
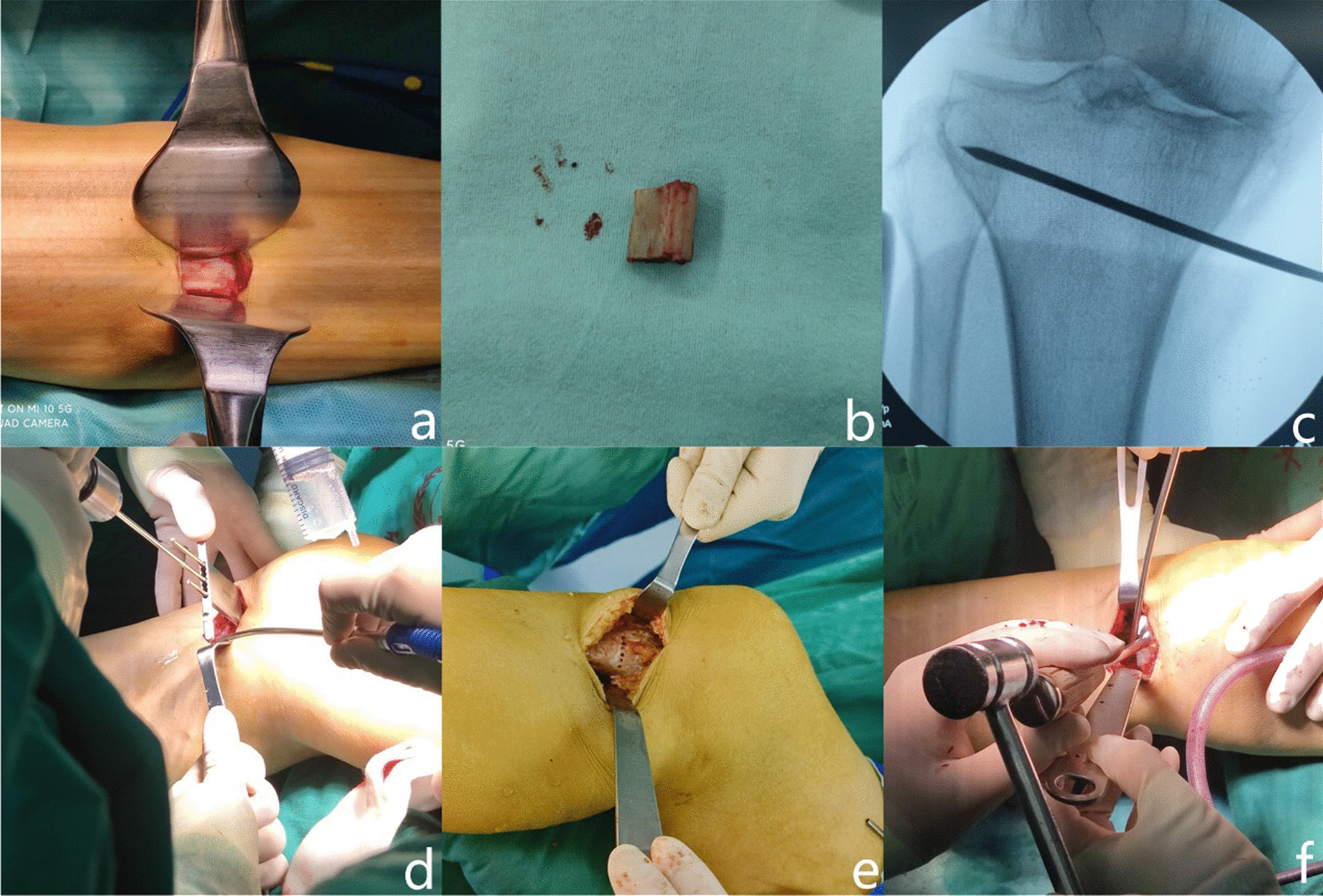
Surgical procedures of spacer-type MOWHTO. The proximal fibular shaft was exposed by splitting the septum between the soleus and peroneus (**a**). The resected proximal fibula (**b**). A 3.5-mm guidewire was inserted 3 cm below the knee joint toward PTFJ to determine the osteotomy plane (**c**). The proximal tibia was drilled on the osteotomy plane with a multihole parallel guiding apparatus and Kirschner wires [(**d**) and (**e**)]. Test models of different thicknesses were hammered into the osteotomy plane (**f**)

**Fig. 2:**
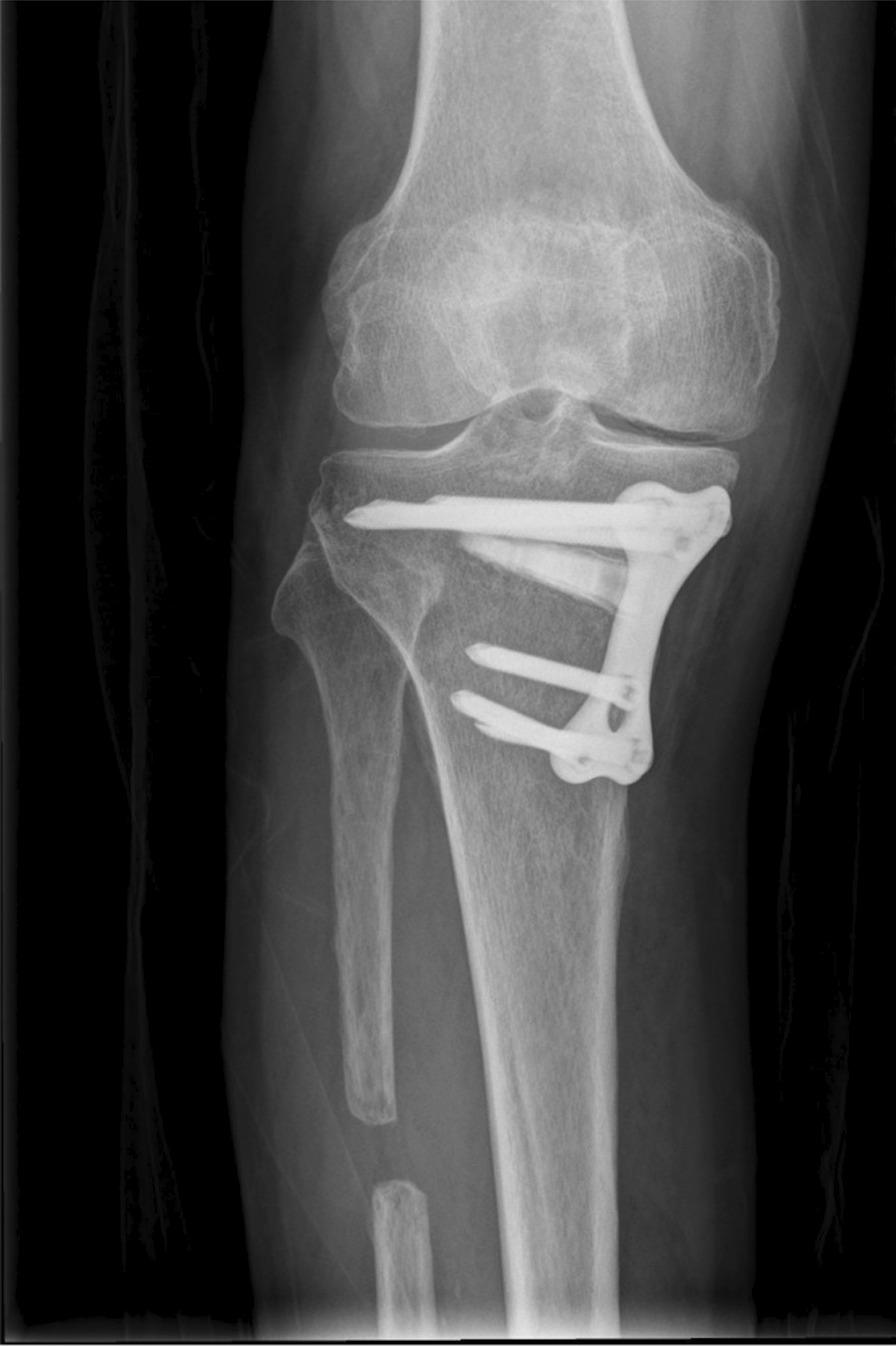
A buttress plate was used for patients with unstable hinge fracture during the surgical procedures

The same doctor performed the operation under general anesthesia. The surgical procedure was composed of two main steps: proximal fibular osteotomy and high tibial osteotomy. Proximal fibular osteotomy: A 3-cm longitudinal incision was made approximately four fingerbreadths below the head of the fibula to avoid iatrogenic injury to the peroneal nerve. The proximal fibular shaft was exposed by splitting the septum between the soleus and peroneus. Then, the fibula with a length of approximately 2 cm was cut using a saw blade, and the peroneal nerve was protected from damage by a retractor (Fig. 1a, b). Finally, the incisions were irrigated with saline. High tibial osteotomy: A 4-cm longitudinal incision was made 2 cm inferior to the knee, which extended along the posterior one-third of the tibia. The periosteum was cut between the patellar tendon and pes anserinus to expose the proximal tibia. A 3.5-mm guidewire was inserted 3 cm below the knee joint toward the proximal tibiofibular joint (PTFJ) to identify the optimal hinge position (Fig. 1c). Then, the proximal tibia was drilled on the osteotomy plane with a multihole parallel guiding apparatus and Kirschner wires (Fig. 1d, e). Through this process, the stiff bones get softened to reduce the rate of unexpected fracture during subsequent surgical procedures. After that, the holes in the tibia were connected using a chisel with scale. A sufficient osteotomy, reaching 10 mm near the lateral tibial cortex, was done on both anterior and posterior cortices toward the level of PTFJ to prevent hinge fracture. Test models of different thicknesses were slowly and carefully hammered into the osteotomy plane (Fig. 1f). Meanwhile, an assistant applied a moderate valgus force on the ipsilateral distal tibia to create an elastic tension. The mechanical axis of the lower extremity was assessed under fluoroscopic guidance to guarantee that the preoperative varus deformity was corrected to a slight valgus or to neutral position. Then, an absorbable spacer implantation with a suitable size was implanted. There were numerous holes and agnails on the absorbable spacer that could accelerate bone union and enhance the stability of the osteotomy plane. If no unstable hinge fracture was found under fluoroscopy, the incisions were sutured after irrigation with normal saline. A buttress plate was needed to stabilize the tibia during the surgical procedures if an unstable hinge fracture was found (Fig. 2).

### Preoperative preparation and postoperative rehabilitation

**Fig. 3:**
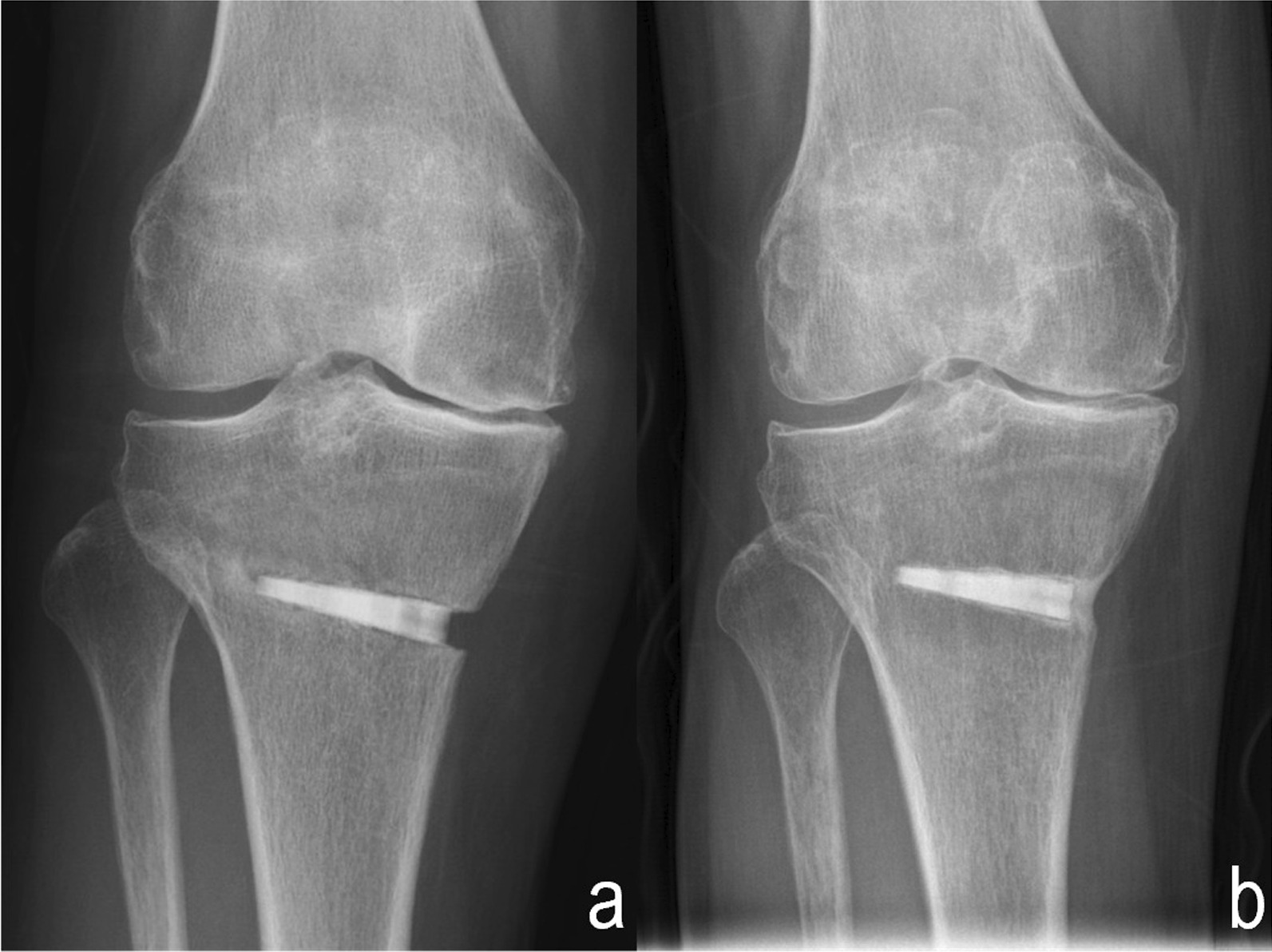
Bone union at the osteotomy site was shown 3 days (**a**) and 4 months after surgery (**b**)

Preoperative physical preparation: All patients completed a 3-week preoperative high-intensity isokinetic resistance training program focusing on quadriceps and hamstring strength. Postoperative rehabilitation: Antibiotics were administered within 24 h after surgery. Patients were encouraged to exercise joint function on the bed to avoid joint stiffness and deep vein thrombosis. The isokinetic resistance training program focusing on the quadriceps and hamstring strength was performed on the duration before full weight-bearing was allowed. Partial weight-bearing with crutches was initiated at 6 weeks postoperatively. Full weight-bearing was allowed when there was evidence of bone union at the osteotomy site (almost 3 months postoperatively) (Fig. 3). For patients with a BMI > 30 or severe osteoporosis, the time to full weight-bearing was delayed.

### Clinical and radiological measurements

Clinical outcomes were evaluated using the Western Ontario and McMaster Universities Arthritis Index (WOMAC) score, which consisted of three subscales: pain, function, and stiffness. The WOMAC scores were evaluated preoperatively and one year postoperatively by two orthopedic surgeons who did not participate in the surgery. According to the postoperative total WOMAC scores, patients were divided into two groups: The patients who had a total WOMAC score ≤ 40 points were classified into the effective group and patients who had a total WOMAC score > 40 points were classified into the invalid group. The method and classification criteria have been described in previous research [20, 21].

**Fig. 4:**
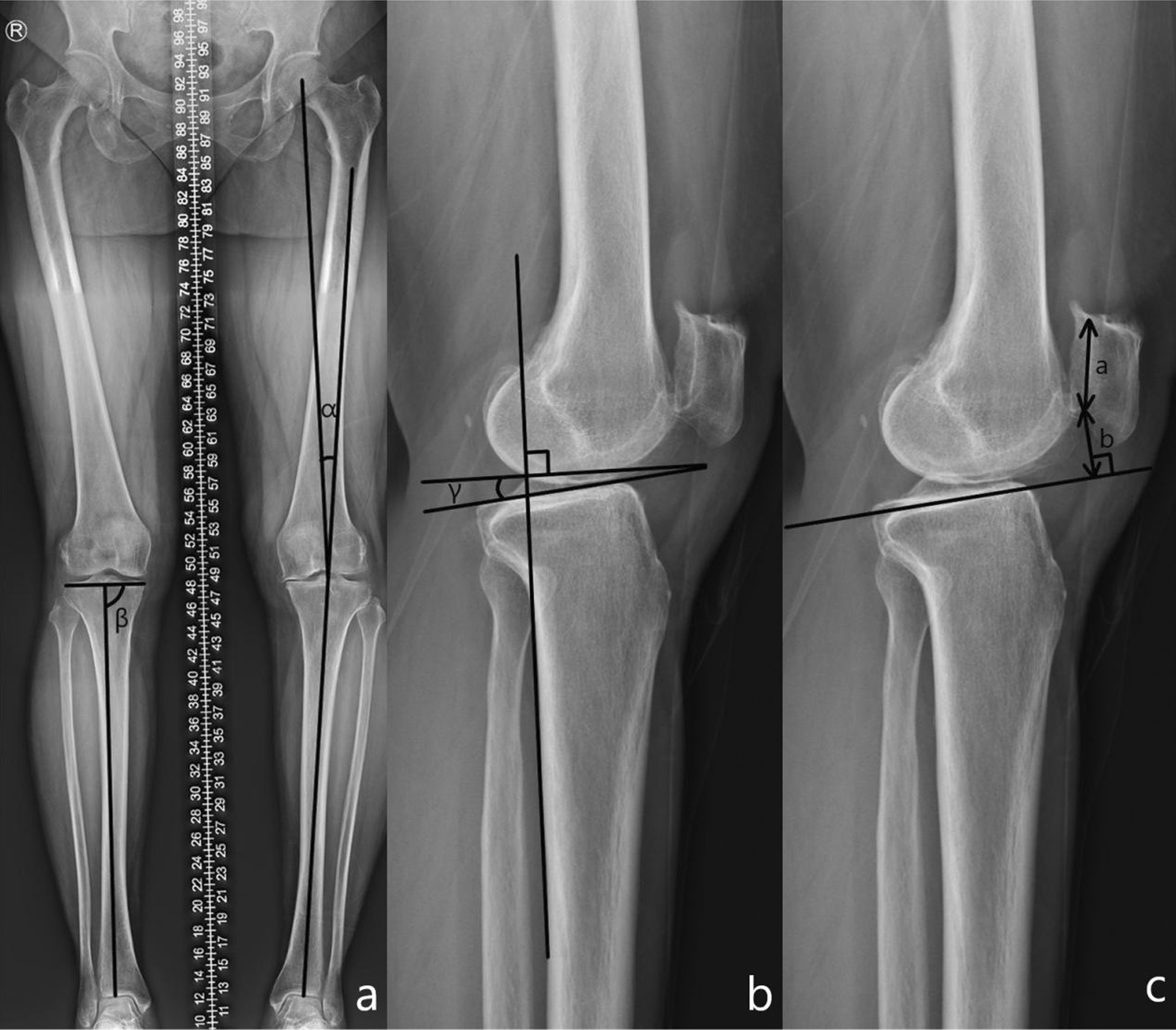
Definition and measurement of HKAA, MPTA, PTSA, and BPI. Angle α expresses the HKAA, angle β expresses the MPTA (**a**), and angle γ expresses the PTSA (**b**). The Blackburn–Peel index (BPI) is equal to b/a (**c**).

A radiological evaluation was performed preoperatively and one year postoperatively (Fig. 4). All radiological data were recorded by a dedicated radiology technician under the supervision of two orthopedic surgeons who did not participate in the surgery. The radiographic evaluations entailed the assessment of full-length standing hip-to-ankle radiographs and anteroposterior and lateral knee radiographs. The medial proximal tibial angle (MPTA), hip–knee–ankle angle (HKAA), posterior tibial slope angle (PTSA), and Blackburn–Peel index (BPI) were measured on the radiographs. The HKAA was defined as the angle between the mechanical axis of the femur and the tibia. The MPTA was defined as the medial angle between the tibial mechanical axis and a line connecting the tibial plateaus. The PTSA was defined as the angle between a line connecting the apex points at the anterior and posterior borders of the lateral tibial plateau and a line perpendicular to the posterior tibial cortex. BPI was defined as the perpendicular distance from the lower margin of the patellar articular surface to the tibial plateau line divided by the length of the patellar articular surface on lateral radiographs.

### Statistical analysis

Normally distributed variables are expressed as the means and standard deviations, and categorical variables are presented as frequencies. The Kolmogorov–Smirnov normality test was employed before statistical analysis to determine whether parametric tests were used. The radiologic measurements between the two groups were compared with independent *t* tests. Analysis of categorical or dichotomized data utilized the chi-squared test. Multiple logistic regression analysis was performed for the variables with a *P* < 0.1 in the univariate analyses. Odds ratios (ORs) and 95% confidence intervals (CIs) were calculated. Intraclass correlation coefficients (ICCs) with 95% CIs were used to evaluate the reproducibility or radiographic measurements, and ICCs > 0.75 were considered to represent excellent agreement. All computations were performed using standard software (SPSS 23.0; SPSS Inc, Chicago, IL); *P* < 0.05 was considered statistically significant.

## Results

### Demographics of patients

A total of 104 patients (36 males and 68 females) with a mean age of 61.67 ± 7.71 years (range 46–78 years) were eligible for our study protocol. Further details of the characteristics of the study cohort and the study protocol are shown in Table 1.

**Table 1:** Patients demographic data

Characteristic	Value
No. of patients	104
Age (y)	61.67 ± 7.71
Male/female	36:68
Body mass index (kg/m^2^)	28.08 ± 3.35
K–L grade II/III/IV	58/30/16
Duration of symptoms (y)	6.85 ± 5.51

Values are shown as mean ± standard deviation

*BMI* body mass index, *K–L grade* Kellgren–Lawrence

### Clinical and radiographic outcomes

**Fig. 5:**
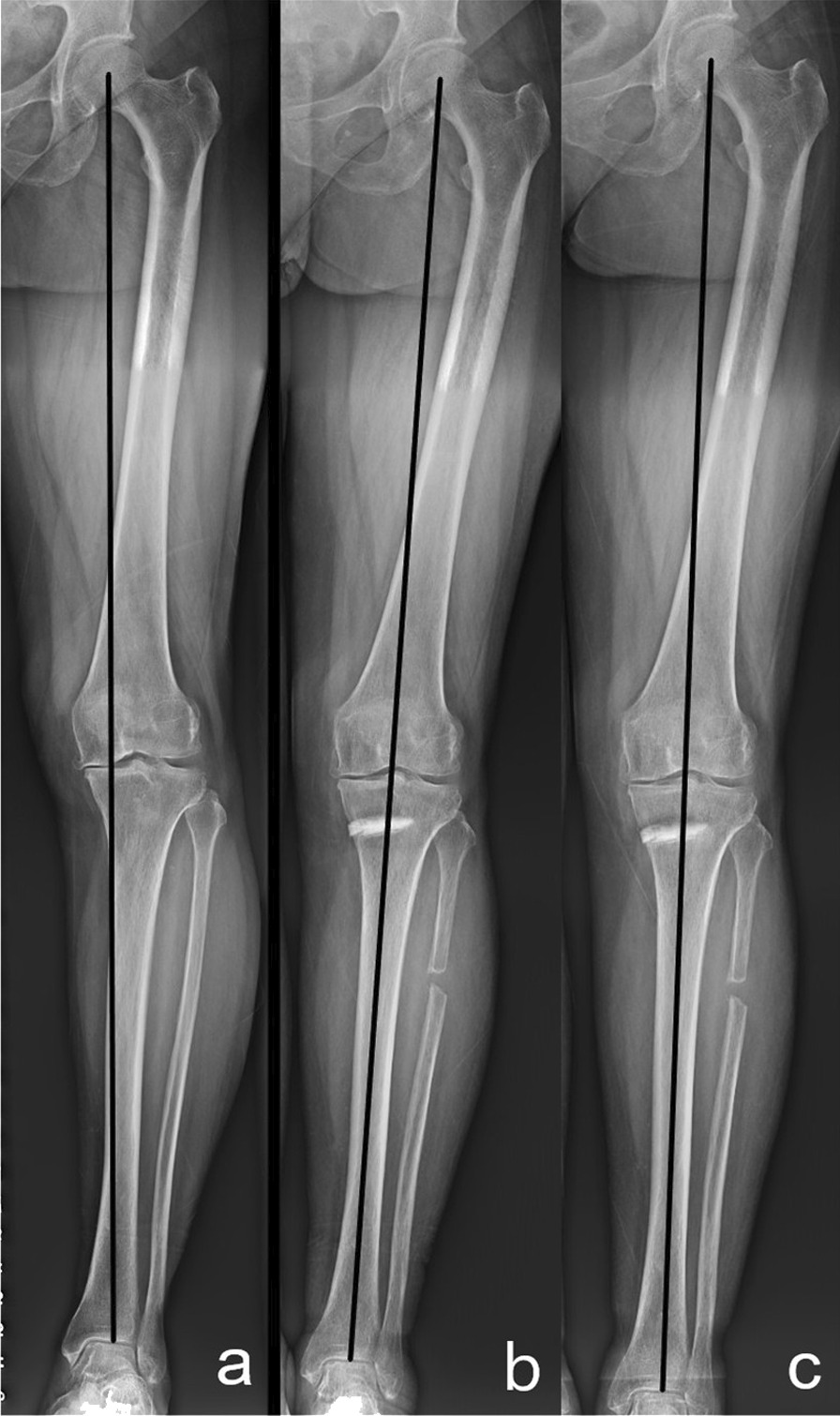
The changes in lower limb alignment preoperatively (**a**), 6 (**b**) and 12 months (**c**) postoperatively

The WOMAC scores were significantly decreased at one year after surgery compared with the preoperative baseline values (*P* < 0.001). According to the classification criteria, 16 (15.4%) patients were classified into the invalid group, and 88 (84.6%) were classified into the effective group. The postoperative HKAA and MPTA were significantly corrected compared with preoperative indicators. All preoperative varus deformities were corrected to a slight valgus or to a neutral position, causing a significant decrease in the varus angle (Fig. 5). Moreover, the PTSA was decreased, and the patellar height was not significantly changed postoperatively (Table 2). For all preoperative and postoperative radiologic measurement data, the reproducibility among radiological values was excellent (ICCs = 0.845–0.922), and the interobserver agreement for K–L grades of OA was strong (*κ* = 0.822).

**Table 2:** Comparison of preoperative and postoperative clinical results and radiologic parameters

	Preoperative	Postoperative 1 year	*P *value
HKAA (°)	7.58 ± 3.28	− 2.05 ± 1.58	< 0.001
MPTA (°)	85.95 ± 2.35	92.31 ± 2.46	< 0.001
PTSA (°)	9.27 ± 3.54	7.33 ± 3.34	< 0.001
BPI	0.75 ± 0.11	0.73 ± 0.13	0.084
WOMAC score	72.39 ± 12.95	20.06 ± 12.96	< 0.001

Values are shown as mean ± standard deviation

*WOMAC* Western Ontario McMaster University Osteoarthritis Index Scales, *HKAA* hip–knee–ankle angle, *PTSA* the posterior tibial slope angle, *MPTA* medial proximal tibial angle, *BPI* the Blackburn–Peel index

### Risk factor analyses of postoperative results at one year

By comparing the variables between the two groups, the mean values of age, BMI, pre-HKAA and percentage of K–L grade IV were significantly higher in the invalid group (Table 3). Univariate analysis revealed that preoperative age > 70, BMI > 30, pre-HKAA > 10° and K–L grade IV were significant predictors for multiple logistic regression analysis (*P* < 0.1 for all) (Table 4). Multivariable logistic regression analysis showed that age > 70 (OR = 4.861) and K–L grade IV (OR = 6.590) were significantly associated with the higher total WOMAC score at one year after surgery (Table 5).

**Table 3:** Comparison of preoperative and postoperative factors between two groups

Factors	Effective group (*n* = 88)	Invalid group (*n* = 16)	*P* value
Age (y)	60.59 ± 6.95	67.63 ± 9.11	0.009*
Sex:female	59 (67%)	9 (56%)	0.238
BMI (kg/m^2^)	27.71 ± 3.43	30.09 ± 1.86	< 0.001*
Duration of symptoms (y)	6.56 ± 5.36	8.40 ± 6.21	0.281
K–L grade			< 0.001*
K–L grade II	54 (61%)	4 (25%)	
K–L grade III	26 (30%)	4 (25%)	
K–L grade IV	8 (9%)	8 (50%)	
Pre-PTSA (°)	9.29 ± 3.52	9.17 ± 3.79	0.906
Pre-HKAA (°)	7.20 ± 3.27	9.72 ± 2.44	0.004*
Pre-MPTA (°)	86.14 ± 2.30	84.91 ± 2.44	0.175
Pre-BPI	0.75 ± 0.11	0.76 ± 0.16	0.706
Pre-WOMAC	72.82 ± 13.55	70.06 ± 8.96	0.436
Post-PTSA (°)	7.24 ± 3.24	7.83 ± 3.89	0.578
Post-HKAA (°)	− 2.12 ± 1.62	− 1.68 ± 1.36	0.262
Post-MPTA (°)	92.26 ± 2.42	92.60 ± 2.76	0.647
Post-BPI	0.74 ± 0.12	0.73 ± 0.18	0.795

Data are presented as means ± SD

*BMI* body mass index, *K–L grade* Kellgren–Lawrence grade, *PTSA* the posterior tibial slope angle, *HKAA* hip–knee–ankle angle, *MPTA* medial proximal tibial angle, *BPI* the Blackburn–Peel index, *WOMAC* Western Ontario and McMaster Universities Osteoarthritis Index score

*P value < 0.1

**Table 4:** Univariate analyses of parameters

Factors	Effective group (*n* = 88)	Invalid group (*n* = 16)	*P* value	Unadjusted ORs	95% CIs	*P* value
*Age (y)*						
≤ 70	78 (89)	9 (56)	0.001*	Ref		
> 70	10 (11)	7 (44)		6.607	1.851–19.882	0.003*
*BMI (kg/m*^*2*^*)*						
≤ 30	67 (76)	8 (50)	0.032*	Ref		
> 30	21 (24)	8 (50)		3.190	1.067–9.544	0.038*
*K–L grade*						
II–III	79 (90)	9 (56)	0.001*	Ref		
IV	9 (10)	7 (44)		6.827	2.047–22.772	0.002*
*Pre-HKAA (°)*						
≤ 10	68 (77)	8 (50)	0.024*	Ref		
> 10	20 (23)	8 (50)		2.667	0.900–7.904	0.077*

Data are presented as *n* (%)

*BMI* body mass index, *K–L grade* Kellgren–Lawrence grade, *HKAA* hip–knee–ankle angle

*P value < 0.1

**Table 5:** Results of multiple logistic regression analysis

Factors		*P* value	OR	95% CI
Age (y)	> 70	0.021*	4.861	1.264–18.695
BMI (kg/m^2^)	> 30	0.113	2.773	0.785–9.801
K–L grade	IV	0.007*	6.590	1.665–27.476
Pre-HKAA (°)	> 10	0.122	2.751	0.762–9.932

Data are presented as *n* (%)

*BMI* body mass index, *K–L grade* Kellgren–Lawrence grade, *HKAA* hip–knee–ankle angle, *OR* odds ratio, *CI* confidence interval

*P value < 0.05

## Discussion

MOWHTO reportedly achieves significant improvements in pain and function, slows down the progression of OA, and postpones the need for TKA [5, 8, 10, 14, 18]. However, this surgical treatment also causes numerous problems during follow-up. First, the treatment effect seems to deteriorate over time, with reported survival rates of 51–97.6% at ten years postoperatively [5, 10, 22–28]. Second, complications occur after MOWHTO in 10–50% of patients [5, 29]. Although there are few serious adverse events, the incidence of complications is higher after MOWHTO (28%) than after TKA (7%) [30, 31]. Third, MOWHTO is not the ultimate treatment for OA of the medial knee compartment, and the outcome of TKA performed after MOWHTO may be influenced by factors such as the patellar height, condylar offset, and/or tibial inclination angle [19]. Therefore, clinical treatment must be guided by strict indications and predictors, especially the novel spacer-type MOWHTO.

Because of the differences between surgical procedures, the prognostic factors of spacer-type MOWHTO may differ from those of conventional MOWHTO with internal fixation [18]. In our study, spacer-type MOWHTO obtained better results in patients with younger age and lower K–L grade, while age > 70 years and K–L grade IV were identified as risk factors for dissatisfaction following spacer-type MOWHTO. Previous studies have shown a correlation between tibial radiological values and clinical signs, but no study has evaluated the effect of these indices on the outcome of spacer-type MOWHTO. The HKAA, MPTA, and PTSA were corrected to a specific high tibial osteotomy standard position to ensure the attainment of anatomic postoperative lower limb alignment and tibial plateau retroversion. Some studies have reported that a small HKAA (< 15°) is more suitable for spacer-type MOWHTO [18, 19]. Therefore, we excluded patients with radiographic measurements that were outside the ranges suggested in the literature. This means that more stringent preoperative planning is needed for spacer-type MOWHTO than for conventional MOWHTO to ensure efficacy and minimize complications.

Although the incidence of knee OA was significantly higher in women than in men, our study found that sex was not a significant prognostic factor for spacer-type MOWHTO [5, 10]. Furthermore, the long-term history did not affect the frequency of poor results.

Age was a significant predictive factor of a poor outcome after spacer-type MOWHTO. For patients older than 70 years, it is difficult to achieve ideal knee functional recovery after surgery. Long-term incomplete weight-bearing (approximately three months) and osteoporosis may be the main causes of muscular atrophy and decreased physical activity [5, 32, 33]. Muscle strength recovery was still a challenge for elderly patients after surgery, even though muscle training was used in patients. Older patients often have more severe osteoporosis, which increases the risk of intraoperative hinge fractures. Advanced age and osteoporosis are also risk factors that affect the healing of the osteotomy plane, which can delay the complete weight-bearing time. Furthermore, the increased recovery time results in activity reduction and increases the risk of lower extremity thrombosis and decreases lung function.

In our study, the outcome of spacer-type MOWHTO was much worse for patients with severe knee OA with a K–L grade of IV than for patients with a K–L grade of II-III. This suggests that the outcome of spacer-type MOWHTO was affected by degeneration of the knee joint. Severe knee lesions such as patellofemoral arthrosis [34], synovitis, cartilage defects, or ligamentous knee instability [34] may have affected the surgery outcome even if lower limb alignment is completely corrected. Similarly, previous studies have reported that patients with K–L grade IV OA achieve unsatisfactory results after conventional HTO. Studies have shown that inflammatory factors in the joints play a more important role than dynamics in the late clinical manifestations of knee OA [35–37].

Although BMI was not considered to be an independent risk factor for spacer-type MOWHTO, we thought that patients with a high BMI had a higher risk of surgical complications. The incidence of complications such as loss of valgus correction angle due to loosening and prolapse of the implantation, hinge fracture, persistent postoperative pain and numbness, and reoperation was higher in patients with a high BMI. However, for conventional MOWHTO, the locking compression plate provides reliable stability that enables the cut tibia to bear body weight [17]. Due to the instability of the medial tibia after spacer-type MOWHTO, the placement of too much weight on the wedge cross end can cause implantation prolapse or hinge fracture.

Compared with traditional MOWHTO, this novel method has many advantages, such as a lower cost, avoiding the need for secondary surgery for fixation removal, maintaining the patellar height to avoid degeneration of the patellofemoral joint [10, 31, 38–40], and decreasing the posterior tibial slope. However, the longitudinal stability of the cut tibia cannot be guaranteed. Patients needed to experience long-term incomplete weight-bearing (approximately 3 months) to guarantee great bone union and stability of the osteotomy plane. This was a shortcoming of this novel method, which may have affected the postoperative results of some patients and caused other complications [41]. It might be expected that long-term incomplete weight-bearing would produce muscular atrophy, loss of proprioception, and decreased physical activity [42]. Therefore, strict surgical indications and appropriate pre- and postoperative rehabilitation plans are essential.

Our study had several limitations that may have influenced the results. First, the follow-up duration was relatively short, with a maximum of 1 year. Thus, the follow-up duration was too short to enable the accurate evaluation of the outcome of the spacer-type MOWHTO but can be used to evaluate the short- or medium-term curative effect. Long-term follow-up studies are needed to confirm the predictive factors affecting the clinical outcomes of spacer-type MOWHTO. Second, fibular osteotomy was performed during spacer-type MOWHTO, which may have impacted the clinical outcomes. However, recent studies have reported good outcomes after fibular osteotomy.

## Conclusions

The results of our study showed that age and K–L grade are the main prognostic factors associated with postoperative outcomes following spacer-type MOWHTO, which supports our hypothesis. Spacer-type MOWHTO seems most suitable for younger patients with less severe OA of the medial compartment. Therefore, to ensure appropriate patient expectations regarding surgical outcomes, these factors should be considered when choosing between spacer-type MOWHTO or conventional MOWHTO. The patients should be fully informed about the advantages and disadvantages of the two surgical procedures.

## Data Availability

All data generated or analyzed during this study are included in this article.

## Abbreviations

MOWHTO: Medial open-wedge high tibial osteotomy
BMI: Body mass index
HKAA: Hip–knee–ankle angle
MPTA: Medial proximal tibial angle
PTSA: Posterior tibial slope angle
BPI: Blackburn–Peel index
WOMAC: Western Ontario and McMasters University Osteoarthritis Index

## References

[1] Woolf AD, Pfleger B (2003). Burden of major musculoskeletal conditions. Bull World Health Organ.

[2] Zhao Y, Zhu Z, Chang J, Wang G, Zheng S, Kwoh CK (2021). Predictive value of the morphology of proximal tibiofibular joint for total knee replacement in patients with knee osteoarthritis. J Orthop Res.

[3] Kim KI, Seo MC, Song SJ, Bae DK, Kim DH, Lee SH (2017). Change of chondral lesions and predictive factors after medial open-wedge high tibial osteotomy with a locked plate system. Am J Sports Med.

[4] Sterett WI, Steadman JR, Huang MJ, Matheny LM, Briggs KK (2010). Chondral resurfacing and high tibial osteotomy in the varus knee: survivorship analysis. Am J Sports Med.

[5] Spahn G, Kirschbaum S, Kahl E (2006). Factors that influence high tibial osteotomy results in patients with medial gonarthritis: a score to predict the results. Osteoarthr Cartil.

[6] van Lieshout WAM, van Ginneken BJT, Kerkhoffs G, van Heerwaarden RJ (2020). Medial closing wedge high tibial osteotomy for valgus tibial deformities: good clinical results and survival with a mean 4.5 years of follow-up in 113 patients. Knee Surg Sports Traumatol Arthrosc.

[7] Koshino T, Wada S, Ara Y, Saito T (2003). Regeneration of degenerated articular cartilage after high tibial valgus osteotomy for medial compartmental osteoarthritis of the knee. Knee.

[8] Jung WH, Takeuchi R, Chun CW, Lee JS, Ha JH, Kim JH (2014). Second-look arthroscopic assessment of cartilage regeneration after medial opening-wedge high tibial osteotomy. Arthroscopy.

[9] Sun X, Wang J, Su Z (2020). A meta-analysis of total knee arthroplasty following high tibial osteotomy versus primary total knee arthroplasty. Arch Orthop Trauma Surg.

[10] Kohn L, Sauerschnig M, Iskansar S, Lorenz S, Meidinger G, Imhoff AB (2013). Age does not influence the clinical outcome after high tibial osteotomy. Knee Surg Sports Traumatol Arthrosc.

[11] Schröter S, Nakayama H, Yoshiya S, Stöckle U, Ateschrang A, Gruhn J (2019). Development of the double level osteotomy in severe varus osteoarthritis showed good outcome by preventing oblique joint line. Arch Orthop Trauma Surg.

[12] Lee OS, Ahn S, Ahn JH, Teo SH, Lee YS (2018). Effectiveness of concurrent procedures during high tibial osteotomy for medial compartment osteoarthritis: a systematic review and meta-analysis. Arch Orthop Trauma Surg.

[13] Kumagai K, Akamatsu Y, Kobayashi H, Kusayama Y, Koshino T, Saito T (2017). Factors affecting cartilage repair after medial opening-wedge high tibial osteotomy. Knee Surg Sports Traumatol Arthrosc.

[14] Harris JD, McNeilan R, Siston RA, Flanigan DC (2013). Survival and clinical outcome of isolated high tibial osteotomy and combined biological knee reconstruction. Knee.

[15] van Wulfften Palthe AFY, Clement ND, Temmerman OPP, Burger BJ (2018). Survival and functional outcome of high tibial osteotomy for medial knee osteoarthritis: a 10–20-year cohort study. Eur J Orthop Surg Traumatol.

[16] Birmingham TB, Giffin JR, Chesworth BM, Bryant DM, Litchfield RB, Willits K (2009). Medial opening wedge high tibial osteotomy: a prospective cohort study of gait, radiographic, and patient-reported outcomes. Arthritis Rheum.

[17] Zaki SH, Rae PJ (2009). High tibial valgus osteotomy using the Tomofix plate–medium-term results in young patients. Acta Orthop Belg.

[18] Zhang R, Li S, Yin Y, Guo J, Chen W, Hou Z (2021). Open-wedge HTO with absorbable β-TCP/PLGA spacer implantation and proximal fibular osteotomy for medial compartmental knee osteoarthritis: new technique presentation. J Invest Surg.

[19] Deng X, Chen W, Zhao K, Zhu J, Hu H, Cheng X (2021). Changes in patellar height and posterior tibial slope angle following uniplanar medial opening wedge high tibial osteotomy using a novel wedge-shaped spacer implanation concurrent with proximal partial fibulectomy. Int Orthop.

[20] Walker LC, Clement ND, Bardgett M, Weir D, Holland J, Gerrand C (2018). The WOMAC score can be reliably used to classify patient satisfaction after total knee arthroplasty. Knee Surg Sports Traumatol Arthrosc.

[21] Tubach F, Ravaud P, Baron G, Falissard B, Logeart I, Bellamy N (2005). Evaluation of clinically relevant states in patient reported outcoms in knee and hip osteoarthritis: the patient acceptable symptom state. Ann Rheum Dis.

[22] Duivenvoorden T, Brouwer RW, Baan A, Bos PK, Reijman M, Bierma-Zeinstra SM (2014). Comparison of closing-wedge and opening-wedge high tibial osteotomy for medial compartment osteoarthritis of the knee: a randomized controlled trial with a six-year follow-up. J Bone Jt Surg Am.

[23] Webb M, Dewan V, Elson D (2018). Functional results following high tibial osteotomy: a review of the literature. Eur J Orthop Surg Traumatol.

[24] Akizuki S, Shibakawa A, Takizawa T, Yamazaki I, Horiuchi H (2008). The long-term outcome of high tibial osteotomy: a ten- to 20-year follow-up. J Bone Jt Surg Br.

[25] Saragaglia D, Blaysat M, Inman D, Mercier N (2011). Outcome of opening wedge high tibial osteotomy augmented with a Biosorb® wedge and fixed with a plate and screws in 124 patients with a mean of ten years follow-up. Int Orthop.

[26] Amendola A, Bonasia DE (2010). Results of high tibial osteotomy: review of the literature. Int Orthop.

[27] Papachristou G, Plessas S, Sourlas J, Levidiotis C, Chronopoulos E, Papachristou C (2006). Deterioration of long-term results following high tibial osteotomy in patients under 60 years of age. Int Orthop.

[28] Sprenger TR, Doerzbacher JF (2003). Tibial osteotomy for the treatment of varus gonarthrosis. Survival and failure analysis to twenty-two years. J Bone Jt Surg Am.

[29] Marti RK, Verhagen RA, Kerkhoffs GM, Moojen TM (2001). Proximal tibial varus osteotomy. Indications, technique, and five to twenty-one-year results. J Bone Jt Surg Am.

[30] Stukenborg-Colsman C, Wirth CJ, Lazovic D, Wefer A (2001). High tibial osteotomy versus unicompartmental joint replacement in unicompartmental knee joint osteoarthritis: 7–10-year follow-up prospective randomised study. Knee.

[31] Brouwer RW, Huizinga MR, Duivenvoorden T, van Raaij TM, Verhagen AP, Bierma-Zeinstra SM (2014). Osteotomy for treating knee osteoarthritis. Cochrane Database Syst Rev.

[32] Hortobágyi T, Garry J, Holbert D, Devita P (2004). Aberrations in the control of quadriceps muscle force in patients with knee osteoarthritis. Arthritis Rheum.

[33] Creamer P, Lethbridge-Cejku M, Hochberg MC (2000). Factors associated with functional impairment in symptomatic knee osteoarthritis. Rheumatology (Oxford).

[34] Rudan JF, Simurda MA (1990). High tibial osteotomy. A prospective clinical and roentgenographic review. Clin Orthop Relat Res.

[35] Meliconi R, Pulsatelli L (2019). Are mechanisms of inflammation joint-specific in osteoarthritis?. Rheumatology (Oxford).

[36] Gessl I, Popescu M, Schimpl V, Supp G, Deimel T, Durechova M (2021). Role of joint damage, malalignment and inflammation in articular tenderness in rheumatoid arthritis, psoriatic arthritis and osteoarthritis. Ann Rheum Dis.

[37] Zheng L, Zhang Z, Sheng P, Mobasheri A (2021). The role of metabolism in chondrocyte dysfunction and the progression of osteoarthritis. Ageing Res Rev.

[38] Krause M, Drenck TC, Korthaus A, Preiss A, Frosch KH, Akoto R (2018). Patella height is not altered by descending medial open-wedge high tibial osteotomy (HTO) compared to ascending HTO. Knee Surg Sports Traumatol Arthrosc.

[39] Gaasbeek RD, Nicolaas L, Rijnberg WJ, van Loon CJ, van Kampen A (2010). Correction accuracy and collateral laxity in open versus closed wedge high tibial osteotomy. A one-year randomised controlled study. Int Orthop.

[40] Brouwer RW, Bierma-Zeinstra SM, van Raaij TM, Verhaar JA (2006). Osteotomy for medial compartment arthritis of the knee using a closing wedge or an opening wedge controlled by a Puddu plate. A one-year randomised, controlled study. J Bone Jt Surg Br.

[41] Lee OS, Ahn S, Lee YS (2017). Effect and safety of early weight-bearing on the outcome after open-wedge high tibial osteotomy: a systematic review and meta-analysis. Arch Orthop Trauma Surg.

[42] Kean CO, Birmingham TB, Garland SJ, Bryant DM, Giffin JR (2011). Preoperative strength training for patients undergoing high tibial osteotomy: a prospective cohort study with historical controls. J Orthop Sports Phys Ther.

